# Circadian Rhythms and Clock Genes in Reproduction: Insights From Behavior and the Female Rabbit’s Brain

**DOI:** 10.3389/fendo.2018.00106

**Published:** 2018-03-15

**Authors:** Mario Caba, Gabriela González-Mariscal, Enrique Meza

**Affiliations:** ^1^Centro de Investigaciones Biomédicas, Universidad Veracruzana, Xalapa, Mexico; ^2^Centro de Investigación en Reproducción Animal, CINVESTAV-Universidad Autónoma de Tlaxcala, Tlaxcala, Mexico

**Keywords:** maternal behavior, lactation, PER1 protein, suckling, pregnancy, parturition, preoptic area, oxytocin

## Abstract

Clock gene oscillations are necessary for a successful pregnancy and parturition, but little is known about their function during lactation, a period demanding from the mother multiple physiological and behavioral adaptations to fulfill the requirements of the offspring. First, we will focus on circadian rhythms and clock genes in reproductive tissues mainly in rodents. Disruption of circadian rhythms or proper rhythmic oscillations of clock genes provoke reproductive problems, as found in clock gene knockout mice. Then, we will focus mainly on the rabbit doe as this mammal nurses the young just once a day with circadian periodicity. This daily event synchronizes the behavior and the activity of specific brain regions critical for reproductive neuroendocrinology and maternal behavior, like the preoptic area. This region shows strong rhythms of the PER1 protein (product of the Per1 clock gene) associated with circadian nursing. Additionally, neuroendocrine cells related to milk production and ejections are also synchronized to daily nursing. A threshold of suckling is necessary to entrain once a day nursing; this process is independent of milk output as even virgin does (behaving maternally following anosmia) can display circadian nursing behavior. A timing motivational mechanism may regulate such behavior as mesolimbic dopaminergic cells are entrained by daily nursing. Finally, we will explore about the clinical importance of circadian rhythms. Indeed, women in chronic shift-work schedules show problems in their menstrual cycles and pregnancies and also have a high risk of preterm delivery, making this an important field of translational research.

## Introduction

**Figure 1:**
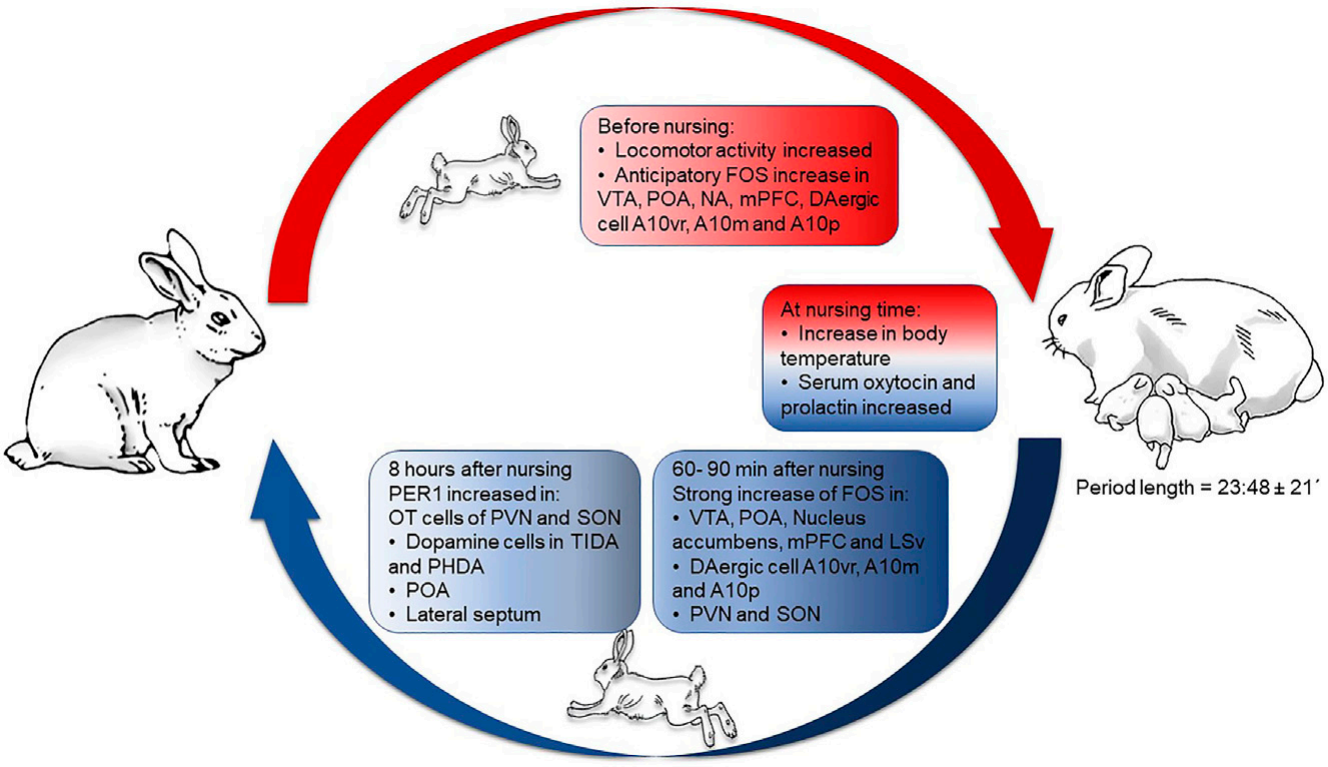
Behavioral, physiological, and neural changes throughout circadian lactation in the rabbit doe. Abbreviations: A10vr, A10 ventral rostral; A10m, A10 medial; A10p, A10 posterior; FOS, c-Fos protein; mPFC, medial prefrontal cortex; NA, nucleus accumbens; OT, oxytocin; PHDA, periventricular hypophysial dopaminergic cells; POA, preoptic area; PVN, paraventricular nucleus of the hypothalamus; SON, supraoptic nucleus; TIDA, tuberoinfundibular dopaminergic cells; VTA, ventral tegmental area. In non-pregnant, non-lactating females FOS protein rhythms reach a peak at different hours in different structures, but in lactating does all of these rhythms shift to the hour of nursing. Figure derived from data previously published in Ref. (44, 49, 62–64, 66, 79, 80).

Few studies have explored the relation between circadian rhythms and reproduction. Most of the early works focused on lactation and maternal behavior (MB), largely in rodents. However, the discovery of functional molecular clock machinery in reproductive tissues, and the use of clock gene mutant models have revealed that such genes play a main role in orchestrating reproductive processes in mammals. First, we will focus on circadian rhythms and clock genes in reproductive tissues, from implantation through lactation, mainly in rodents. Then, we will focus on the rabbit, a lagomorph with a striking circadian rhythm of lactation, unique to this class of mammals. Our studies in this animal are revealing, entraining of behaviors and neuroendocrine processes in specific brain structures as a consequence of suckling by pups (Figure 1). Finally, we will explore the translational importance of a “healthy” circadian clock for proper rhythms in reproduction.

## Circadian Rhythms and Clock Genes in Reproductive Processes

Many physiological processes and behaviors in mammals are rhythmic. The most evident daily change is the sleep/wake cycle, but there are clear changes in the blood concentration of several hormones and specific metabolites throughout the day (1). These changes allow organisms to adapt to the environmental light/dark cycle and consequently to the resources available at specific times of day or night. These rhythms are controlled by an endogenous molecular clock within the suprachiasmatic nucleus (SCN), located in the forebrain of mammals, which is entrained by the light/dark cycle. The molecular clockwork is composed of a group of core clock genes, *Per, Cry, Clock*, and *Bmal1*, organized in a transcription–translation feedback loop that oscillates every 24 h. Their oscillations are associated with self-sustaining redox rhythms, known as nontranscriptional clocks as well as metabolic rhythms in an organ-specific manner [Reviewed in Ref. (1)]. Reproductive tissues have also functional molecular clocks and, although at the top of the hierarchy are the SCN oscillations, it is now recognized that the circadian system is organized along several axes of a redundant network that exchanges bidirectional timing information among the components (2, 3). An early study found that lesions to the SCN completely eliminated phasic LH release (4), and in recent years much information has accumulated to support the importance of the clockwork mechanism in reproduction by using mutant mouse models with various disruptions of the molecular clockwork. Recently, in *Clock/Clock* mutant mice it was demonstrated that few of these animals became pregnant, they had a high rate of fetal reabsorption and severe dystocia and the fetuses showed morphological abnormalities (5, 6). However, it is possible that this is an effect not only of the *Clock/Clock* mutation as *Per1, Per2*, and *Bmal1* knockout mice, but also shows several abnormalities during pregnancy and parturition (7, 8). Very little is known about the possible mechanisms involved. In *Clock/Clock* mutants, serum progesterone levels are twofold lower and estradiol is significantly lower in mid-pregnancy compared to wild-type females, differences that have been associated with a high incidence of pup reabsorption (5). Indeed, impaired steroidogenesis appears to be a common problem in clock gene mutants as pregnant *Bmal1* (−/−) mice also have lower progesterone serum levels than *Bmal1* (+/±) and reduced embryo implantation (9). Moreover, in rats, deletion of ovarian *Bmal1* gene affected genes critical for progesterone production, leading to implantation failure; these effects were reversed by the implantation of a single wild-type ovary (10). Regarding *Per1* and *Per2* mutants, although fertile, they exhibit lower reproductive success than the control group, as occurs in aged wild-type mice (7). Together, the above information indicates that proper oscillations of the core clock genes in reproductive tissues are necessary for successful ovulation, embryo implantation, and steroidogenesis (11). In Table 1, we summarize some effects on reproduction provoked by alterations in specific clock genes. These reproductive disorders are observed in clock gene-deficient animals. Thus, it remains to be determined at which specific levels of control clock genes act, as the functions described in Table 1 are complex and have a multifactorial regulation. Moreover, as clock genes control transcription in a tissue-specific manner and recently nontranscriptional metabolic clocks have been discovered [Reviewed in Ref. (1)], the possibility exists that endocrine factors (i.e., specific hormones) could play a main role in the expression of reproductive disorders related to clock gene disruption.

**Table 1:** Some effects in reproductive success by changes in clock genes genotype in mammals.

Clock gene	Species	Effect	Reference
**Gonads**			
*Bmal 1^−/−^*	Mouse	Ovarian size reduced	(8)
*Bmal 1*^−/−^	Mouse	Low testosterone and high luteinizing hormone in serum; reduction in esteroidogenic genes in testes, reduced sperm count. Infertility	(12, 13)
*Clock*	Human polymorphism	Semen volume reduction, low sperm motility, and idiopathic infertility. Alteration in serum levels of testosterone and FSH	(14, 15)
*Cry1*	Mouse KD	Reduction of meiotic process and maturation in oocytes	(16)
*Bmal1^flx/flx^*	Mouse	Changes in phasic LH sensitivity of theca cells in ovary	(17)
*Bmal1^flx/flx^*	Mouse	Failure to mate with receptive females. Low secretion of FSH and GnRH. Tyrosine hydroxylase in brain decreased	(18)

**Estrous and menstrual cycles**			
*Clock^Δ19^*	Mouse	Higher proportion of irregular estrous cycles	(19–21)
*Clock*	Mouse^clock/clock^	Irregular estrous cyclicity and failure to have a coordinated LH surge on proestrus	(22)
*Bmal 1*^−/−^	Mouse KO	Changes in daily pattern of estrogen receptor β in tissues implicated in female reproductive functions	(23, 24)
*Clock*	Human polymorphism	Irregular menstrual cycles	(25)

**Gestational/parturition**			
*Per 1*^−/−^ and *Per 2*^−/−^	Mouse	Successful parturition reduced	(7)
*Bmal 1*^−/−^	Mouse	Lack of implantation and embryonic development. Impaired steroidogenesis, low progesterone levels and embryo implantation reduced. Alterations in delivery times	(8–10, 26)
*Clock*	Mouse^clock/clock^	Elevated rates of fetal reabsorption	(5)
*Bmal 1*	Human polymorphism	Miscarriages increased	(27)

**Postpartum success**			
*Per 1*^−/−^ and *Per 2*^−/−^	Mouse KO	Number of pups weaned reduced	(7)
*Clock^Δ19^*	Mouse	Postnatal mortality increased and low prolactin levels and reduced milk production	(19, 28)

In rats, delivery occurs at daytime, i.e., during the rest period [Reviewed in Ref. (29)], and destruction of the SCN disrupts the timing of birth (30). Takayama et al. (31) explored the role of the pineal gland hormone melatonin (MEL) and found that pinealectomized rats gave birth at either day or night and that MEL replacement at night (but not during the day), across pregnancy, restored the timing of parturition during the day in most subjects. Interestingly, in rodents, the placenta expresses functional clock genes and also glucocorticoid receptors (32) and MEL receptor MT1 (33), which are rhythmically expressed. Thus, it is possible that maternal central hormonal secretions also drive the activity of the placenta in pregnancy and parturition (34). By contrast, in primiparous rabbits kept under laboratory conditions (14 h light:10 h dark) parturition occurs throughout the day, regardless of litter size delivered (35).

Regarding lactation, mother rats nurse more often during the resting phase, i.e., across daytime (36, 37). In mice, maternal crouching (nursing posture) peaks during the day and is less frequent during the night and, concomitantly, prolactin serum levels are higher during the day (28). By contrast, *Clock* mutant mice do not have a significant peak of either crouching or prolactin, and the amount of milk secreted from mutant mice is lower (as calculated by a significant lower body weight of pups) when compared to wild-type dams (28). Additionally, pups from homozygous *Bmal1* null mice are 30% lighter at weaning (8), supporting the importance of a circadian molecular clock in timing MB and lactation. In cows, the mammary gland’s demand for nutrients in early lactation is several-fold increased over that seen during pregnancy and this demand is not met just by increasing food intake (38), a finding from which a compensatory circadian mechanism was proposed. During the transition from pregnancy to lactation, there is an upregulation of the positive limb of the core clockwork as well as of clock regulatory genes in specific metabolic pathways of the rat’s mammary gland, liver, and adipose tissues to support the increased nutritional demands of lactation [Reviewed in Ref. (39)]. Accordingly, in mice *Per1* and *Bmal1*, mRNA levels are elevated in late pregnant and lactating mammary tissues supporting their role in mammary gland development and differentiation (40).

## Nursing Within a Circadian Context: The Rabbit Model

Doe rabbits nurse the young once a day, for approximately 3 min, inside a nest constructed by the mother across pregnancy (41). This invariability in the nursing pattern is observed throughout lactation (ca. 30 days), despite a marked increase in milk output across the first 20 days and a gradual decline thereafter (42). Nursing occurs at night, under light:dark or continuous light conditions, with circadian periodicity (43, 44). A threshold of suckling stimulation is essential for this regulation as reducing litter size below six kits disrupts the circadian expression of nursing (35). Although deliveries occur throughout the day, a population of parturient rabbits becomes synchronized to initiate and maintain nursing at around the same time from lactation day 1 onward. A Rayleigh analysis of the hour of nursing in the population of studied does indicated that, despite the hour of delivery most nursing episodes occurred during the night, at 03:51 h, from postnatal days 1–15 (35). This adjustment is possible because a negative correlation exists between time of delivery and time of nursing on lactation day 1, i.e., mothers giving birth in the early morning show longer “parturition-nursing” intervals than does delivering at later hours.

A normal duration of nursing bouts also depends on a threshold of suckling as mothers given four kits or less spend longer times inside the nest box (45). Yet, milk output *per se* is not essential to display a normal nursing *behavior* as virgins induced to behave maternally (by lesioning the main olfactory system) can enter the nest box, crouch over the litter, allow suckling, and exit ca. 3 min later. This behavioral pattern is observed with circadian periodicity in 55% of maternal virgins (46).

## PER1 Protein Rhythms Shift by the Timing of Nursing

Suckling induces oxytocin (OT) secretion in all mammals and, in rabbits, the amount secreted is directly related with the number of suckling kits (47). Does OT participate in translating the suckling stimulus received at the nipple to the brain regions regulating nursing periodicity and duration? The number and size of OT-immunoreactive (IR) neurons increases in the paraventricular hypothalamic nucleus (PVN) from estrus, through pregnancy, and into lactation (48). Following suckling, the total number of c-FOS-IR cells increases significantly in this structure (49). Bilateral lesions to the PVN of lactating rabbits abolish or disrupt the circadian display of nursing, but do not modify duration of suckling bouts (50). Although in rabbits no OT receptors are evident in the PVN, they are abundant in the prefrontal cortex, preoptic area (POA), and lateral septum [LS (51)], regions that participate in regulating specific aspects of the doe’s MB (52, 53).

The doe’s circadian nursing pattern is, in turn, a timing signal for the kits (54). By scheduling the hour of nursing we have shown that this predictable event entrains rhythms of locomotor behavior, metabolic parameters, plasma corticosterone hormones, and also several brain structures in 7–9-day-old kits (55–57). From these findings, we proposed that rabbit kits are a natural model of food entrainment (57, 58). The synchronization of brain structures was determined by quantifying the expression of the PER1 protein, product of the *Per1* clock gene. The rhythm of this protein can be synchronized to a particular stimulus, e.g., food cues, in specific brain regions (59). Thus, while the clockwork oscillations of the SCN are synchronized to the light/dark cycle, the rhythm of clock genes in peripheral tissues and in the brain can be entrained by stimuli other than light, like food (60, 61). From the findings that: (a) single or multiple entrances to the nest depend on the number of suckling kits (35, 45); (b) preventing suckling by kits on lactation days 7–9 significantly decreased the amount of PER1 protein at peak time in both PVN and supraoptic nucleus (62, 63), we consider that suckling can be an entraining signal for PER1 protein rhythms on particular neuroendocrine populations, specifically oxytocinergic and also in dopaminergic (DAergic) cells. Thus, in estrous does maintained under light:dark conditions [12:12; lights on at 07:00 = time (ZT) 0], PER1 protein in the PVN peaks at ZT15, as occurs in tyrosine hydroxylase (TH)-IR cells that co-express PER1. By contrast, in lactating rabbits the peak of PER1 and PER1/TH appears 4 h after the timing of scheduled nursing. DAergic populations from the tuberoinfundibular and periventricular hypophysial regions, related to the control of prolactin release in the hypophysis, also shift their rhythm of co-expression with PER1 protein according to the timing of suckling. In contrast, no change was observed in incertohypothalamic DAergic cells, which are not related to the control of prolactin secretion (63). Therefore, our results suggest that periodic suckling is a time signal for the synthesis and/or secretion of OT and prolactin at a predictable time.

The daily spontaneous return of the mother to the nest coincides with an increase in locomotor behavior (62), suggesting that she is in a state of high arousal to access the kits. Indeed, DAergic cells of the A10 mesolimbic system increase their cellular activity, anticipating daily nursing, supporting the assumption that she is in a high motivational state to visit the kits for nursing (64). Moreover, timing the suckling stimulus also synchronizes the POA and LS, essential for the expression of MB (65), as indicated by rhythms of PER1 (66). These results, together with those of the mesolimbic system (64), suggest the establishment of a “maternal entrainable circuit” where suckling seems to be the entraining signal. Taken together, the entraining of PER1 oscillations points to the importance of the *Per1* gene in specific brain regions for uncoupling their oscillations from the master clock to fulfill a specific reproductive demand, the care, and nourishment of the litter.

## Translational Importance of Circadian Rhythms and Clock Genes Disruption

Disruption of circadian rhythms has profound consequences in humans. Light during the day is the main synchronizer for our circadian rhythms and controls the timing of our neuroendocrine system. For example, the hormone melatonin is secreted only during the night and seems to be a humoral entraining signal for peripheral organs to show proper circadian rhythms (1). Epidemiological studies were the first to indicate that the exposure to artificial light during the night, which disrupts the normal secretion of melatonin (67), is associated with circadian disruptions and to breast cancer [Reviewed in Ref. (68)]. Regarding reproduction, women shift-workers (in which the master clock is exposed to artificial light at night) have an increased risk of endometriosis, irregular menstrual cycles (with pain and unusual menstrual bleeding), delayed ovulation, increased miscarriage rate, preterm delivery, and infant low birth weight (69, 70). It has also been proposed that MEL can be a zeitgeber for the timing of parturition in women (29). The above evidence highlights the importance of central signals from the master clock and pineal MEL to peripheral reproductive organs for proper fetus development, as shown in rats (71). Besides, other organs (e.g., placenta) may play a direct role. Full-term placenta expresses circadian rhythms of *Clock* and *Bmal1* (72), and clock gene polymorphisms are associated with placental abruption (73) and even a single polymorphism of *Bmal1* is associated with an increase in miscarriages (27). Finally, RNA microarray analysis of human milk fat globules indicates differential daily expression of 7% of transcripts (74). Moreover, there are daily changes in the concentration of antibodies and complement proteins of the immune system among several other cellular and soluble components of human milk (75). Interestingly, baby milk formula and food enriched with tryptophan (a precursor of MEL) helps to improve infant sleep when consumed at night (76, 77). This is an emerging area of research known as “chrononutrition” (78).

## Conclusion

Clock genes in reproductive tissues, together with those in the SCN and other brain structures, play a central role in orchestrating circadian rhythms in all reproductive processes from implantation to lactation. Lesion studies of the SCN as well as alterations of the molecular clockwork using mutant mice models have revealed multiple disruptions in all reproductive processes. In contrast, very little is known about circadian rhythms and reproduction in wild-type animals, except in the rabbit. This species offers an extraordinary opportunity for exploring this issue, particularly during lactation as, in lagomorphs, nursing usually occurs once a day with circadian periodicity, a unique characteristic among mammals. Consequently, it is possible to explore in neuroendocrine cells of this species the relevance of particular components of the circadian clockwork with minimal manipulations to the animals, as opposed to rodents, that nurse several times a day. The translational importance of circadian rhythms in reproduction was first recognized through studies of women in shift-work and recently through the finding of differences in the components of breast milk across the circadian cycle, results that could improve the health and well-being of infants.

## Author Contributions

MC, GG-M, and EM contributed to the writing of the manuscript and approved the final version.

## Conflict of Interest Statement

The authors declare that the research was conducted in the absence of any commercial or financial relationships that could be construed as a potential conflict of interest.
